# The impact of repeated vaccination on influenza vaccine effectiveness: a systematic review and meta-analysis

**DOI:** 10.1186/s12916-018-1239-8

**Published:** 2019-01-10

**Authors:** Lauren C. Ramsay, Sarah A. Buchan, Robert G. Stirling, Benjamin J. Cowling, Shuo Feng, Jeffrey C. Kwong, Bryna F. Warshawsky

**Affiliations:** 1 Public Health Ontario, 480 University Avenue Suite 300, Toronto, Ontario M5G 1V2 Canada; 2 Dalla Lana School of Public Health, University of Toronto, 155 College St, Toronto, Ontario M5T 3M7 Canada; 3 Public Health Agency of Canada, 130 Colonnade Road, Ottawa, Ontario K1A 0K9 Canada; 4 WHO Collaborating Centre for Infectious Disease Epidemiology and Control, School of Public Health, Li Ka Shing Faculty of Medicine, The University of Hong Kong, Pokfulam, Hong Kong, China; 5 Institute for Clinical Evaluative Sciences, Veterans Hill Trail, 2075 Bayview Avenue G1 06, Toronto, Ontario M4N 3M5 Canada; 6 Department of Family & Community Medicine, University of Toronto, 155 College St, Toronto, Ontario M5T 3M7 Canada; 7 University Health Network, 399 Bathurst St, Toronto, Ontario M5T 2S8 Canada; 8 Department of Epidemiology and Biostatistics, Western University, 1151 Richmond St, London, Ontario N6A 3K7 Canada

**Keywords:** Influenza, Vaccine effectiveness, Repeated vaccination

## Abstract

**Background:**

Conflicting results regarding the impact of repeated vaccination on influenza vaccine effectiveness (VE) may cause confusion regarding the benefits of receiving the current season’s vaccine.

**Methods:**

We systematically searched MEDLINE, Embase, PubMed, and Cumulative Index to Nursing and Allied Health Literature from database inception to August 17, 2016, for observational studies published in English that reported VE against laboratory-confirmed influenza for the following four vaccination groups: current season only, prior season only, both seasons, and neither season. We pooled differences in VE (∆VE) between vaccination groups by influenza season and type/subtype using a random-effects model. The study protocol is registered with PROSPERO (registration number: CRD42016037241).

**Results:**

We identified 3435 unique articles, reviewed the full text of 634, and included 20 for meta-analysis. Compared to prior season vaccination only, vaccination in both seasons was associated with greater protection against influenza H1N1 (∆VE = 25%; 95% CI 14%, 35%) and B (∆VE = 18%; 95% CI 3%, 33%), but not H3N2 (∆VE = 7%; 95% CI – 7%, 21%). Compared to no vaccination for either season, individuals who received the current season’s vaccine had greater protection against H1N1 (∆VE = 62%; 95% CI 51%, 70%), H3N2 (∆VE = 45%; 95% CI 35%, 53%), and B (∆VE = 64%; 95% CI 57%, 71%). We observed no differences in VE between vaccination in both seasons and the current season only for H1N1 (∆VE = 3%; 95% CI – 8%, 13%), but less protection against influenza H3N2 (∆VE = − 20%; 95% CI – 36%, − 4%), and B (∆VE = − 11%; 95% CI – 20%, − 2%).

**Conclusions:**

Our results support current season vaccination regardless of prior season vaccination because VE for vaccination in the current season only is higher compared to no vaccination in either season for all types/subtypes, and for H1N1 and influenza B, vaccination in both seasons provides better VE than vaccination in the prior season only. Although VE was lower against H3N2 and B for individuals vaccinated in both seasons compared to those vaccinated in the current season only, it should be noted that past vaccination history cannot be altered and this comparison disregards susceptibility to influenza during the prior season among those vaccinated in the current season only. In addition, our results for H3N2 were particularly influenced by the 2014–2015 influenza season and the impact of repeated vaccination for all types/subtypes may vary from season to season. It is important that future VE studies include vaccination history over multiple seasons to evaluate repeated vaccination in more detail.

**Electronic supplementary material:**

The online version of this article (10.1186/s12916-018-1239-8) contains supplementary material, which is available to authorized users.

## Background

Seasonal influenza vaccination is the predominant strategy for preventing influenza-related morbidity and mortality. Annual vaccination is recommended because of waning immunity and because influenza strains undergo antigenic drift, necessitating reviewing and, in most seasons, changing the vaccine to better match the upcoming season’s strains [1]. Because of the frequently changing vaccine, influenza vaccine effectiveness (VE) is assessed annually.

With increasing numbers of people being immunized against influenza annually, the impact of repeated vaccination has gained significant interest. Of particular interest are older adults (65 years and older), who tend to have more comorbidities as they age, as both age and co-morbidities increase their risk of influenza-associated complications [2]. If repeated vaccination negatively impacts current VE, then having been repeatedly vaccinated in earlier years may be detrimental to the protection of older adults when they need it most. Studies from the 1970s and 1980s found inconsistent results regarding the impact of repeated vaccination [3, 4]. In 1999, a systematic review and meta-analysis of field studies, trials, and serologic studies found no evidence of negative impacts of repeated vaccination [5]. More recently, some studies have found VE to be reduced in those who received repeated prior influenza vaccinations [6–8].

Since most VE studies now report estimates taking into account vaccination status for both current and prior seasons, we sought to evaluate the impact of repeated vaccination on VE through a systematic review and meta-analysis. We aimed to assess the impact of repeated vaccination to provide evidence to support patient and clinician decision-making about receiving the current season’s influenza vaccine. We considered two patient-relevant scenarios: (1) for someone who received last season’s vaccine, should he/she also receive this season’s vaccine? (vaccination in both seasons versus prior season only); and (2) for someone who did not receive last season’s vaccine, should he/she receive this season’s vaccine? (vaccination in current season only versus neither season). We also considered a policy-relevant scenario, comparing VE for vaccination in both seasons versus the current season only. This latter scenario is not relevant to patients because they cannot alter their vaccination history; however, the findings may influence policy decisions regarding whether or not to offer annual vaccination to the entire population if there was evidence suggesting that repeated vaccination could negatively impact future VE.

## Methods

### Search strategy and selection criteria

We searched MEDLINE, Embase, PubMed, and Cumulative Index to Nursing and Allied Health Literature (CINAHL) databases from inception to August 17, 2016. We developed a unique search strategy for each database with the assistance of a scientific librarian; across all databases, search terms included “influenza,” “immunization,” “vaccine,” and “effectiveness,” and articles were restricted to those published in English (Additional file 1). Two reviewers (SB, LR) independently screened titles and abstracts, and hand-searched the references of the included articles.

Eligible studies used observational study designs (e.g., prospective cohort, test-negative case-control) and reported VE against medically attended, laboratory-confirmed influenza for four mutually exclusive vaccination groups: current season only, prior season only, both current and prior seasons, and neither season (reference group). Prior season vaccination referred primarily to vaccination status in the year immediately prior to the season being examined. Studies with other definitions of prior season (e.g., any dose in the prior two seasons) were excluded from the meta-analysis, but were described in a qualitative synthesis. We excluded interim VE reports that were superseded by end-of-season reports, and conference abstracts and proceedings. We followed the Preferred Reporting Items for Systematic Reviews and Meta-Analyses (PRISMA) guidelines for reporting results [9].

### Risk of bias assessment

We used the Newcastle-Ottawa scale (NOS) to assess the risk of bias of included case-control and cohort studies [10]. Two reviewers (SB, LR) independently evaluated the quality of each study based on the domains of selection, comparability, and either exposure (for case-control studies) or outcome (for cohort studies). For studies using the test-negative design, we determined whether calendar time had been included in the adjusted analyses [11]. Studies were categorized as being at low, moderate, or high risk of bias if they were missing ≤ 1 item, 2–3 items, or > 3 items on the NOS, respectively [12]. Any disagreements between the two reviewers were resolved by consensus.

### Data analysis

Two reviewers (SB, LR) abstracted the data using a structured electronic data extraction form, extracting study characteristics (e.g., study design, recruitment setting, case definition) and VE estimates for the four vaccination groups, with discrepancies adjudicated by consensus. Whenever possible, we extracted VE reports by influenza type/subtype and age group provided in the articles and only included the most specific results reported (e.g., by age group or influenza type/subtype) in the meta-analysis. Because specific lineage information for influenza B was often unavailable, we used overall estimates for influenza B.

For each study included in the meta-analysis, VE estimates for current season only, prior season only, and both current and prior seasons were assessed against the reference group who were not vaccinated in either season. In the present study, VE estimates from each study were compared for those vaccinated in both the current and prior seasons to those vaccinated in the prior season only and to those vaccinated in the current season only by subtracting the VE estimates. The absolute differences in VE (ΔVE) was stratified by influenza type/subtype and season and calculated as follows: (a) vaccinated in both seasons compared to the prior season only (ΔVE = VE_both_ − VE_prior only_) and (b) vaccinated in both seasons compared to the current season only (ΔVE = VE_both_ − VE_current only_). In both of the above scenarios, ΔVE greater than zero implies a higher VE estimate when vaccinated in both seasons than in either the current or the prior season only. We also assessed the VE of those vaccinated in the current season only compared to those vaccinated in neither season (pooled VE_current only_).

We calculated confidence intervals for ΔVE by bootstrapping using 1000 samples [13]. Similar to previous work [14], we took 1000 samples from VE_current only_, VE_prior only_, and VE_both_. We then estimated 1000 measures of ΔVE for both ΔVE = VE_both_ − VE_current only_ and ΔVE = VE_both_ − VE_prior only_; the 2.5% and 97.5% percentiles for ΔVE were computed as the confidence intervals. We used a random effects model to pool ΔVE estimates to compare the overall difference between vaccination in both seasons with vaccination in either the prior season only or the current season only. To compare VE for those vaccinated in the current season versus those vaccinated in neither season, we used a random effects model to pool the log odds ratio of the current season only VE estimates and converted the final pooled estimate back to a measure of VE. Statistical heterogeneity was assessed using the *I*^2^ statistic and Cochran’s *Q* test. Meta-analyses were performed in MetaXL (Version 2.2, EpiGear International Ltd., Queensland, Australia) with bootstrapping procedures and figures produced in R (Version 3.3.1, R Foundation for Statistical Computing, Vienna, Austria).

## Results

The results presented below have been corrected from our previously published review on this topic. The previous article included one study that did not meet the inclusion criteria, omitted one estimate from an included study, and included two estimates from populations that were already included in other estimates (i.e., estimates from a subset of a population that were also captured in other included estimates).

**Fig. 1:**
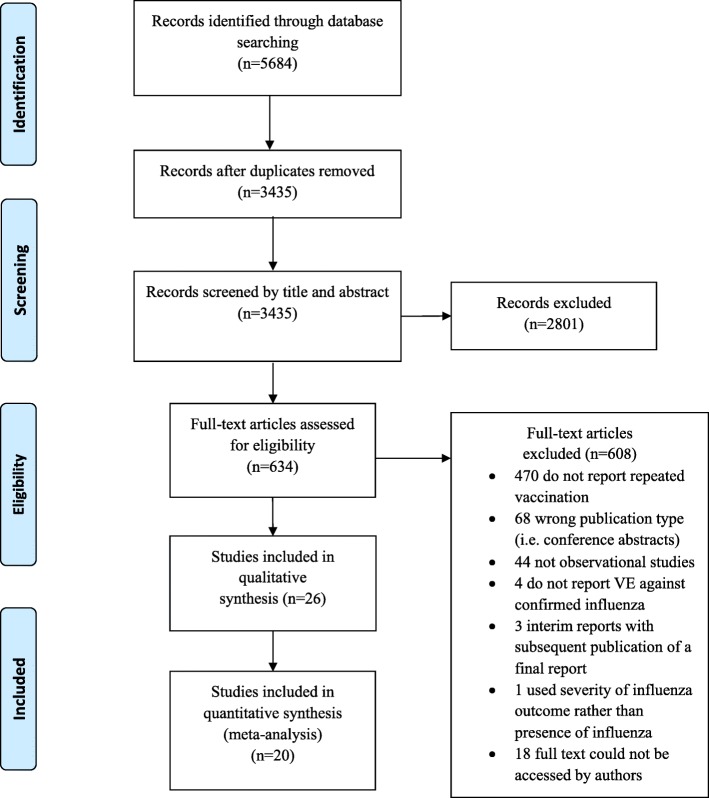
PRISMA flow diagram of study selection

We identified 3435 unique articles from the database searches (Fig. 1). After screening titles and abstracts, we selected 634 articles for full-text review. Of these, 26 studies met the inclusion criteria for the qualitative synthesis, and 20 were included in the meta-analysis [6–8, 15–37]. We observed excellent agreement between reviewers for the title and abstract screen (kappa, *Ƙ* = 0.94) and for the full-text review (*Ƙ* = 0.98). No additional studies were identified from hand-searching references. One study was excluded from the qualitative synthesis and meta-analysis because, while it included persons with laboratory-confirmed influenza in the vaccination groups of interest, the study provided VE estimates only for severe or fatal influenza outcomes rather than for any laboratory-confirmed influenza [38]. We excluded six studies from the meta-analysis but included them in the qualitative synthesis: four studies because they only provided VE estimates for any influenza rather than by influenza type/subtype [15, 23, 31, 32], and two studies because they provided VE estimates for any influenza and “prior season vaccination” was not restricted to the immediate year prior to the study season [16, 26].

The 26 included studies captured influenza seasons between 2004–2005 and 2014–2015, with most reporting estimates for the 2010–2011 to 2014–2015 seasons (Table 1). One study was from the Southern hemisphere [32], one study was restricted to pregnant women [35], and two studies were in pediatric populations [17, 34]. Most studies featured outpatient data, but two studies used solely inpatient data [25, 37], and two studies used data from both settings [15, 16]. All studies used reverse-transcriptase polymerase chain reaction (RT-PCR) testing to confirm influenza infection.

**Table 1:** Study characteristics of articles included in the meta-analysis and/or qualitative synthesis

Author, publication year	Country	Study design	Current season	Prior season	Influenza type	Age group
Jimenez-Jorge et al., 2012 [19]	Spain	Test-negative case-control	2010–2011	2009–2010	H1N1	All ages
Martinez-Baz et al., 2013 [20]	Spain	Test-negative case-control	2010–2011	2009–2010	H1N1	All ages
Skowronski et al., 2012 [28]	Canada	Test-negative case-control	2010–2011	2009–2010	H1N1	All ages
Syrjanen et al., 2014 [33]	Finland	Cohort	2010–2011	2009–2010	H1N1	18–75 years
Fu et al., 2015 [17]	China	Case-control	2012–2013	2011–2012	H1N1	(a) 20–35 months, 1 current dose; (b) 20–35 months, 2 current doses; (c) 3–6 years
Gaglani et al., 2016 [18]	USA	Test-negative case-control	2013–2014	2009–2010 to 2012–2013	H1N1	≥ 9 years
Ohmit et al., 2016 [22]	USA	Prospective cohort study	2013–2014	2012–2013	H1N1	(a) 9 and older; (b) under 9 years
Thompson et al., 2014^b^ [35]	USA	Case-control	2010–2011 and 2011–2012	2009–2010 and 2010–2011	H1N1, H3N2, B	Mean age 30 years
Skowronski et al., 2014b [29]	Canada	Test-negative case-control	2011–2012	2010–2011	H1N1, H3N2, B	≥ 2 years
Rondy et al., 2015 [25]	France, Italy, Lithuania, Spain	Test-negative case-control	2012–2013	2011–2012	H1N1, H3N2, B	≥ 18 years
Skowronski et al., 2014a [27]	Canada	Test-negative case-control	2012–2013	2011–2012	H1N1, H3N2, B	≥ 2 years
Valenciano et al., 2016 [36]	Germany, Hungary, Ireland, Italy, Poland, Portugal, Romania, Spain	Test-negative case-control	2014–2015	2013–2014	H1N1, H3N2, B	All ages
Pebody et al., 2013 [24]	UK	Test-negative case-control	2010–2011	2009–2010	H1N1, B	All ages
Skowronski et al., 2015 [30]	Canada	Test-negative case-control	2013–2014	2012–2013	H1N1, B	≥ 2 years
McLean et al., 2014 [6]	USA	Test-negative case-control	2004–2005 to 2012–2013	Variable	H3N2, B	(a) 9 to 49; (b) 50 and older
McLean et al., 2015 [21]	USA	Test-negative case-control	2012–2013	2011–2012	H3N2, B	(a) 9 to 17; (b) 18 to 49; (c) 50 to 64; (d) 65 and older
Thompson et al., 2016 [34]	USA	Test-negative case-control	2011–2012 and 2012–2013	2010–2011 and 2011–2012	H3N2, B	2–8 years, 1 dose prior season
Skowronski et al., 2016 [7]	Canada	Test-negative case-control	2014–2015	2013–2014	H3N2, B	≥ 2 years
Simpson et al., 2015^a^[26]	Scotland	Test-negative case-control	2008–2009	9 prior seasons	All influenza	All ages
Castilla et al., 2011^a^ [15]	Spain	Nested test-negative case- control	2010–2011	2009–2010	All influenza	All ages
Ohmit et al., 2014 [8]	USA	Test-negative case-control	2011–2012	2010–2011	H3N2	≥ 9 years
Ohmit et al., 2015^a^ [23]	USA	Prospective cohort study	2012–2013	2011–2012	All influenza	All ages
Smithgall et al., 2016^a^ [31]	USA	Surveillance	2013–2014	2012–2013	All influenza	All ages
Castilla et al., 2016^a^ [16]	Spain	Test-negative case-control	2014–2015	2013–2014 and 2012–2013	All influenza	All ages
Sullivan and Kelly, 2013^a^ [32]	Australia	Re-analysis	(a) Southern Hemisphere 2011; (b) Southern Hemisphere 2012	Southern Hemisphere 2010 and 2011	All influenza	All ages
Petrie et al., 2016 [37]	USA	Test-negative case-control	2014–2015	2013–2014	H3N2	≥ 18 years

^a^Study not included in meta-analysis

^b^Study population included pregnant women only

For 24 of the 26 studies, we extracted the variables included in the multivariable regression models used to obtain VE estimates (Additional file 2: Table S1); the remaining two studies did not clearly report these variables. All 24 studies with available information adjusted for age, and the majority adjusted for presence of high-risk conditions or comorbidities (*n* = 17; 71%) and calendar time (*n* = 15; 63%). Many studies also adjusted for time between illness onset and sample collection (*n* = 12; 50%), and sex (*n* = 10; 42%).

All of our included test-negative design studies were deemed to be at low risk of bias, and all included calendar time in their adjusted models [6–8, 15, 16, 18–21, 24–30, 32, 34, 36, 37]. The remaining case-control studies were also categorized as being at low risk of bias [17, 35], as were all the included cohort studies [22, 23, 31, 33]. Details of the evaluation of the included studies are provided in Additional file 3: Figure S1.

**Fig. 2:**
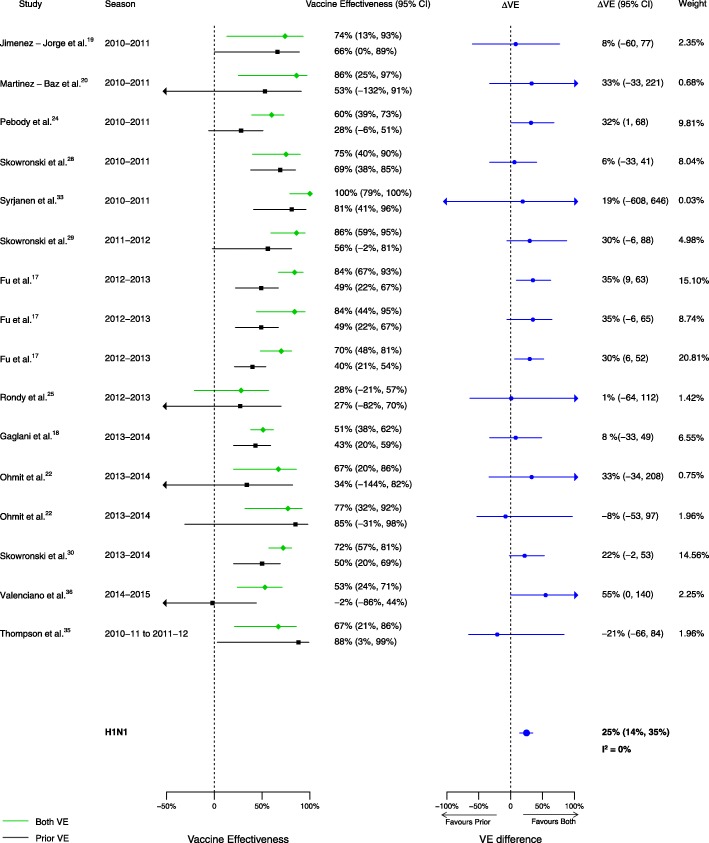
Comparison of VE estimates against influenza H1N1 in those vaccinated in both seasons versus those vaccinated in the prior season only

**Fig. 3:**
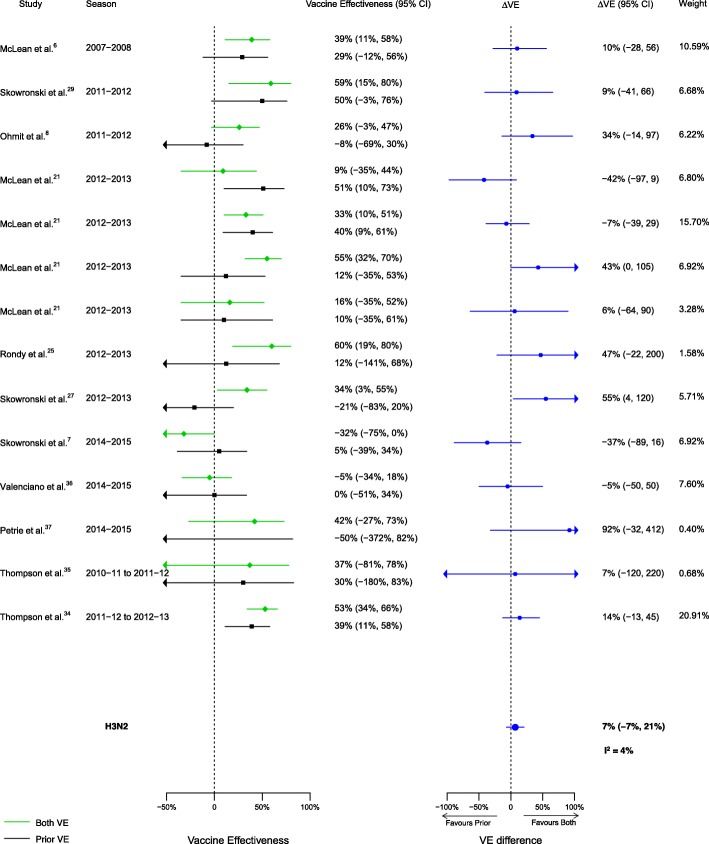
Comparison of VE estimates against influenza H3N2 in those vaccinated in both seasons versus those vaccinated in the prior season only

**Fig. 4:**
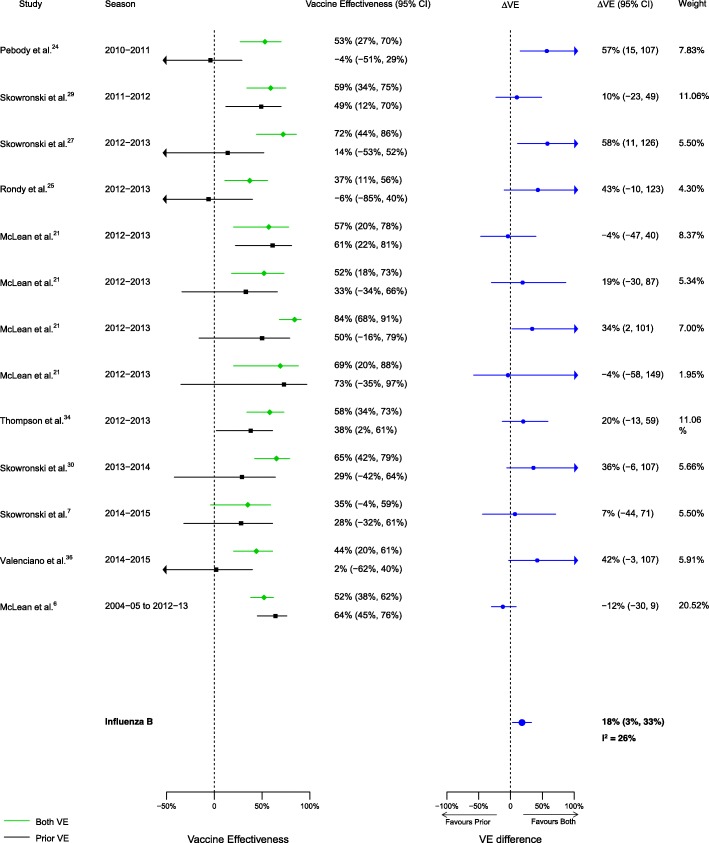
Comparison of VE estimates against influenza B in those vaccinated in both seasons versus those vaccinated in the prior season only

Among the 20 articles included in the meta-analysis, there were 16 analyses for influenza H1N1, 14 for H3N2, and 13 for B that compared VE among those vaccinated in both seasons to those vaccinated in the prior season only. When compared to vaccination in the prior season only, VE was higher for vaccination in both seasons for influenza H1N1 (∆VE = 25%; 95% CI 14%, 35%; *I*^2^ = 0%) and B (∆VE = 18%; 95% CI 3%, 33%; *I*^2^ = 26%), but not H3N2 (∆VE = 7%; 95% CI – 7%, 21%; *I*^2^ = 4%) (Table 2, Figs. 2, 3, and 4). When stratified by influenza season, the results for all seasons were consistent with the overall results (Additional file 4: Table S2).

**Table 2:** Comparison of vaccine effectiveness (VE) by vaccination group and influenza type/subtype

Vaccine effectiveness comparison	Relevance of results	H1N1	H3N2	B
Vaccinated both seasons versus vaccinated prior season only ΔVE^a^ (95% CI) ΔVE = VE_both_ − VE_prior only_	Patient and policy perspectives	*25% (14%, 35%)*	7% (− 7%, 21%)	*18% (3%, 33%)*
Vaccinated current season only versus vaccinated neither season (reference group) Pooled VE_current only_	Patient and policy perspectives	*62% (51%, 70%)*	*45% (35%, 53%)*	*64% (57%, 71%)*
Vaccinated both seasons versus vaccinated current season only ΔVE^a^ (95% CI) ΔVE = VE_both_ – VE_current only_	Policy perspective	3% (− 8%, 13%)	*– 20% (− 36%, − 4%)*	*– 11% (− 20%, − 2%)*

Italic type-face indicates significant results

^a^ΔVE > 0 implies higher vaccine effectiveness estimate when vaccinated in both seasons

**Fig. 5:**
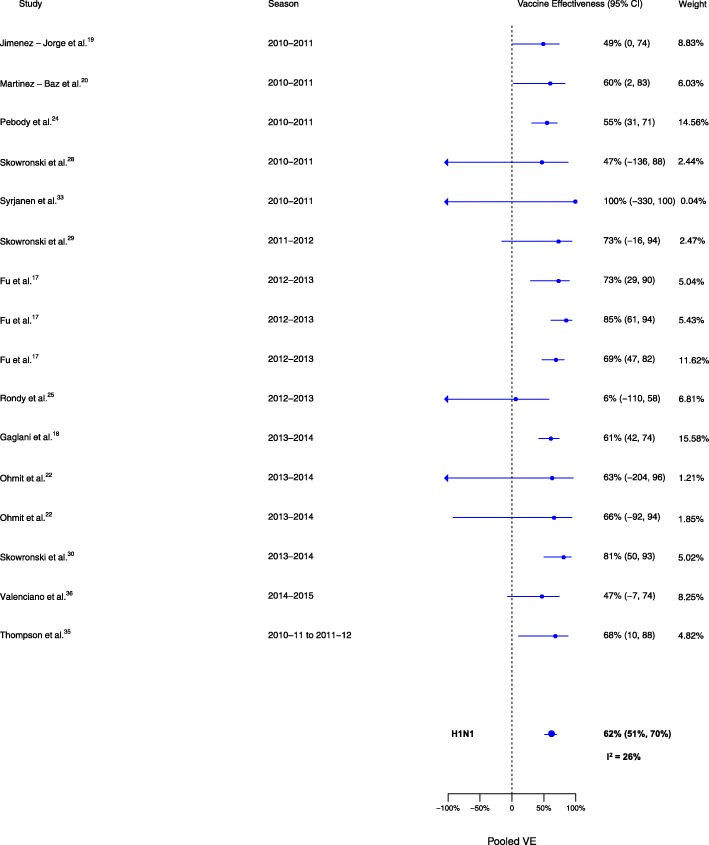
Comparison of VE estimates against influenza H1N1 in those vaccinated in the current season only versus those vaccinated in neither season

**Fig. 6:**
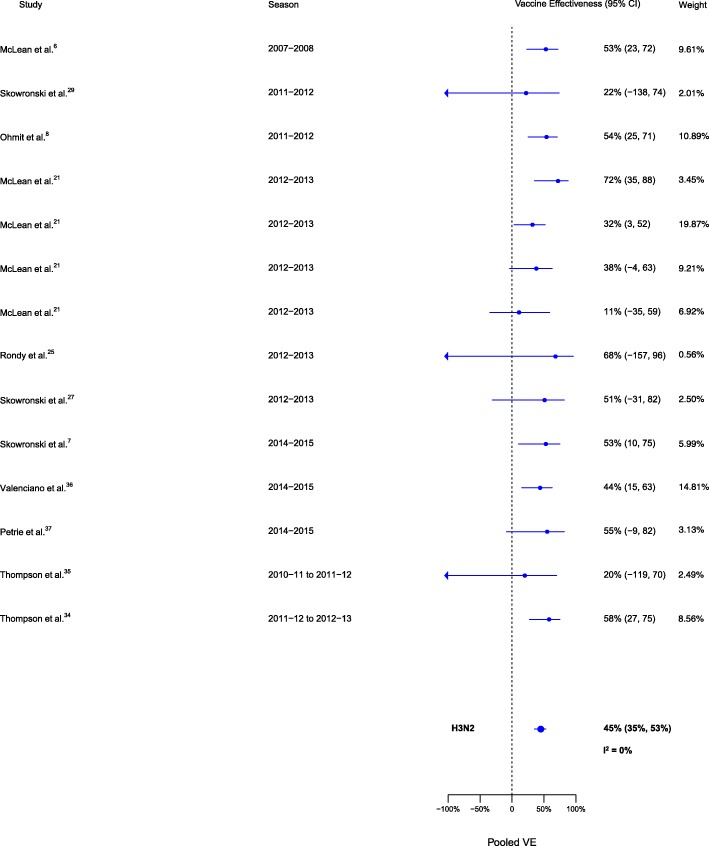
Comparison of VE estimates against influenza H3N2 in those vaccinated in the current season only versus those vaccinated in neither season

**Fig. 7:**
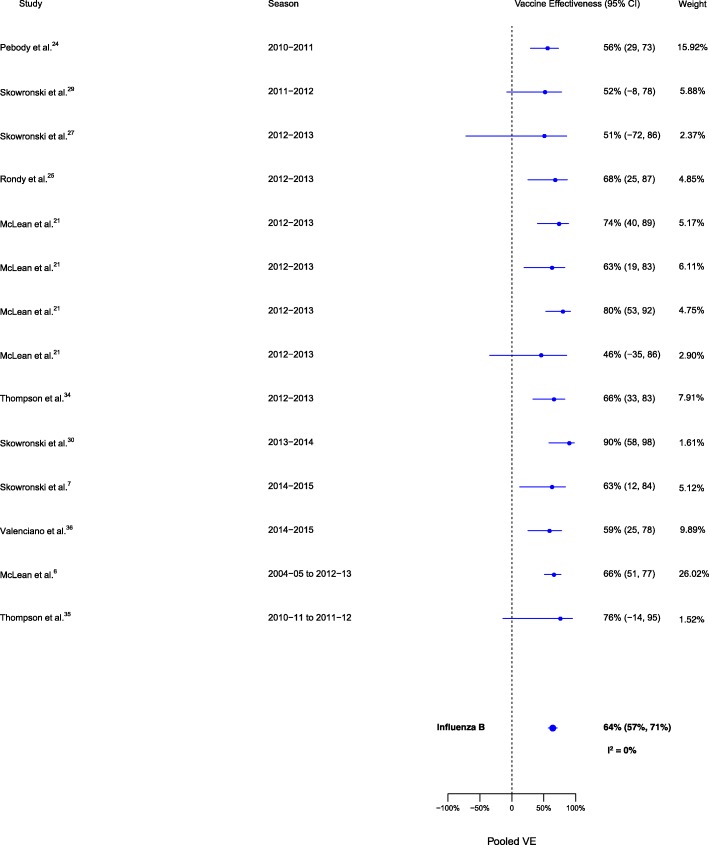
Comparison of VE estimates against influenza B in those vaccinated in the current season only versus those vaccinated in neither season

Sixteen analyses for influenza H1N1, 14 for H3N2, and 14 for B compared VE among those vaccinated in the current season only to those vaccinated in neither season. VE was higher for vaccination in the current season compared to neither season for influenza H1N1 (∆VE = 62%; 95% CI 51%, 70%; *I*^2^ = 26%), H3N2 (∆VE = 45%; 95% CI 35%, 53%; *I*^2^ = 0%), and B (∆VE = 64%; 95% CI 57%, 71%; *I*^2^ = 0%) (Table 2, Figs. 5, 6, and 7). The results for individual seasons were consistent with the overall results (Additional file 4: Table S2).

**Fig. 8:**
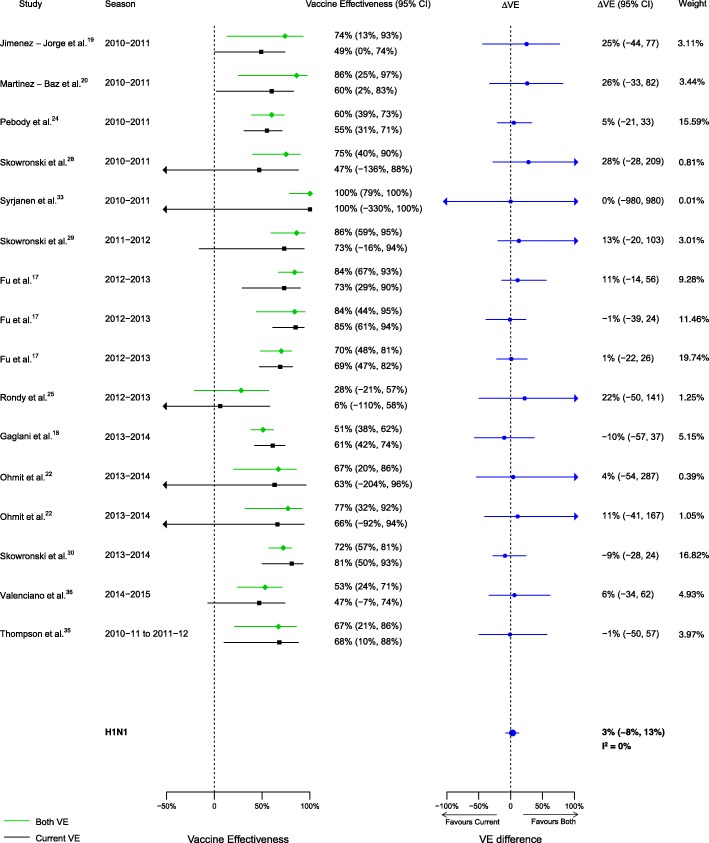
Comparison of VE estimates against influenza H1N1 in those vaccinated in both seasons versus those vaccinated in the current season only

**Fig. 9:**
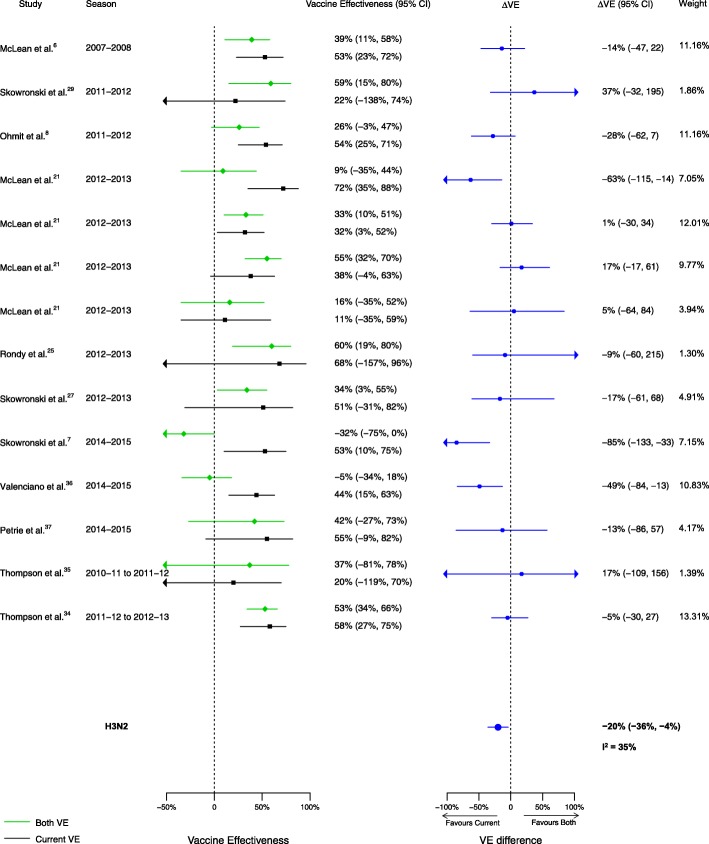
Comparison of VE estimates against influenza H3N2 in those vaccinated in both seasons versus those vaccinated in the current season only

**Fig. 10:**
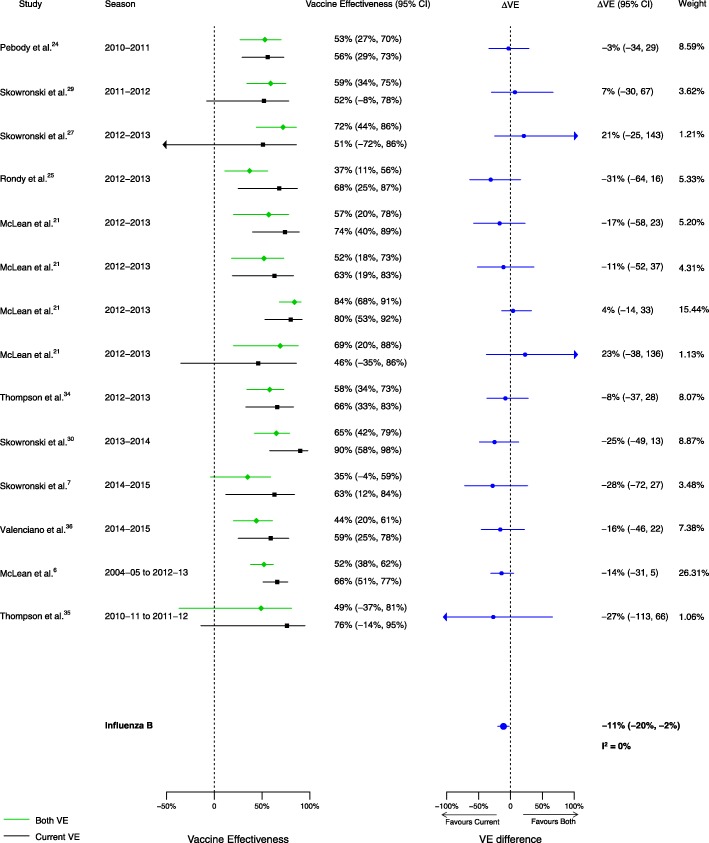
Comparison of VE estimates against influenza B in those vaccinated in both seasons versus those vaccinated in the current season only

For the comparison of those vaccinated in both seasons to those vaccinated in the current season only, 16 analyses for influenza H1N1, 14 for H3N2, and 14 for B compared VE among from the 20 included articles. We observed no statistically significant VE difference between vaccination in both seasons and vaccination in the current season only for influenza H1N1 (∆VE = 3%; 95% CI – 8%, 13%; *I*^2^ = 0%). We observed statistically significant VE differences between vaccination in both seasons and vaccination in the current season only for influenza H3N2 (∆VE = − 20%; 95% CI – 36%, − 4%; *I*^2^ = 35%), and B (∆VE = − 11%; 95% CI – 20%, − 2%; *I*^2^ = 0%) (Table 2, Figs. 8, 9, and 10). For the H3N2 comparison, the results for individual seasons were inconsistent with the overall result except for the 2014–2015 season (i.e., ∆VEs were not statistically significant in the comparison of both seasons versus current season for any season except for 2014–2015) (Additional file 4: Table S2).

To address overlapping patients in the 2010–2011 season analyses in Spain (one conducted in the Navarra region [20] and one that included eight regions in Spain [19]), we performed a sensitivity analysis removing the Navarra region study [20] from the H1N1 meta-analyses and found that it had no effect on the results for any of the three comparisons. When we removed the Navarra region study, we found that for H1N1 VE_both_-VE_prior only_ was 25% (95% CI 14%, 35%); VE_current only_ was 62% (95% CI 51%, 71%); and VE_both_-VE_current only_ was 2% (95% CI− 9%, 13%). The original estimates for H1N1 were 25% (95% CI 14%, 35%), 62% (95% CI 51%, 70%), and 3% (95% CI− 8%, 13%), respectively.

Among the studies included in the qualitative synthesis but not the meta-analysis, two studies presented results using a definition of “prior season vaccination” that included multiple prior seasons and therefore did not meet the inclusion criteria for the meta-analysis [16, 26]. One of these studies considered vaccination history over two consecutive seasons using data from nine influenza seasons (2000–2001 to 2008–2009); those vaccinated in the current season only had the highest VE [26]. Finally, a study from the Navarra region in Spain that assessed vaccination over the current and two prior seasons showed a range of results [16]. Residual VE without current vaccination was noted if vaccinated in both the prior two seasons. For both influenza H3N2 and B, vaccination in the current season and one prior season resulted in quite low VE, whereas vaccination in the current and both prior seasons resulted in higher VE. VE against influenza B was highest among those vaccinated in the current season only compared to the other vaccination groups, whereas this group had the lowest VE against H3N2 [16].

Six studies [15, 16, 23, 26, 31, 32] presented results for any influenza rather than by influenza type/subtype, two of which were summarized above because they also used multiple prior seasons [16, 26]. There were five estimates from the four remaining studies not summarized above. Of these, three favored vaccination in the current season only, and two favored vaccination in both seasons. None of the estimates favored vaccination in the prior season only. In the one study that presented two VE estimates, seasonal differences were apparent. In the Southern Hemisphere 2011 season, the highest VE was observed among those who had been vaccinated in both current and prior seasons, but in the Southern Hemisphere 2010 season, the highest VE was observed among those who had received the current season vaccine only [32].

## Discussion

We found that irrespective of a patient’s vaccination status for the prior season, current season vaccination is associated with greater protection against laboratory-confirmed infection by influenza H1N1 and B. This was evident comparing vaccination in both seasons to vaccination in the prior season only. Furthermore, compared to no vaccination for either season, individuals who received the current season’s vaccine had greater protection against all three influenza types/subtypes. Therefore, vaccination in the current season is generally the best option for the patient. Recent studies have raised questions about the impact of repeated vaccination [6–8], which is of concern to policy-makers with regard to annual influenza vaccination recommendations. Of relevance to the policy-maker (but not of pragmatic relevance to the patient, who cannot alter his/her vaccination history or predict future influenza strain circulation), we observed lower VE in those vaccinated in both seasons compared to only in the current season for influenza H3N2 and B, but not for H1N1. The 2014–2015 influenza season influenced the overall significance, where pooled VE across three studies was lower for those vaccinated in both the current and prior season compared to those vaccinated in the current season alone. However, this comparison does not account for increased susceptibility to influenza during the prior season due to being unvaccinated for that season. Based on the NOS, we assessed that the studies included in this review had a low risk of bias. However, the theoretical underpinnings of the test-negative design are still in the process of explication [39–41], and there has not yet been a theoretical assessment of the potential biases in evaluation of repeated vaccine effects using the test-negative design.

The results of this review are not consistent with those found by Beyer and colleagues in 1999 [5]; their meta-analysis of seven field studies and 12 serologic studies found no significant difference between the single and multiple vaccination groups for all influenza subtypes combined. Our study differs from Beyer et al. by including studies that feature contemporary laboratory testing methods, influenza subtype-specific analysis, and study designs with consistent vaccination comparison groups. A recently published meta-analysis reported pooled VE estimates for the same vaccination status groups as our study (prior only, current only, both seasons) [42]. Similar to our study, that study found VE to be consistently lowest among those vaccinated during the prior season only. As well, for the 2014–2015 season, VE against H3N2 tended to be higher for those vaccinated in the current season only compared to those vaccinated during both seasons. However, that study did not examine the differences in VE as presented in this study.

In our review, the comparison groups used in the meta-analysis provided a more refined calculation of VE that accounted for recent vaccination history. Standard VE calculations (those that do not account for prior vaccination history of the vaccinated group) are comparing those vaccinated in the study season (a mixture of subjects vaccinated in the current season only and those with current and prior vaccination) to a reference group of those not vaccinated in the study season (which includes both those vaccinated in neither season and those vaccinated in the prior season only). Our study allowed for these vaccination groups to be analyzed separately to understand the impact of prior season’s vaccination on current season VE.

Our study was further strengthened by aligning the VE comparisons with patient and policy perspectives, in order to aid decision-making by patients, practitioners, and policy-makers. Additionally, by calculating the differences in VE between the various vaccination groups within each study, we controlled for any methodological biases unique to a particular study, such as study design or study settings (e.g., outpatient vs. hospital-based studies), since these biases would apply equally to each vaccination group. Thus, rather than first pooling the VE estimates from each vaccination group across studies and subsequently taking the difference, we pooled the differences obtained from VE estimates within each study. Finally, because VE can vary by age group and influenza type/subtype, this study was strengthened by the detailed stratification of results by type/subtype, as well as by using VE estimates for the most specific patient groups available (e.g., age-stratified groups rather than “all ages”).

This study also has some limitations. First, the analysis accounts only for vaccination status in one prior season. Results might differ when considering a patient’s vaccination history over a greater number of seasons, which is particularly important when considering the importance of influenza VE in older adults who potentially have received many years of consecutive vaccinations. McLean and colleagues found no difference when exploring VE over two consecutive seasons, but when they used a reference group with no vaccination over six seasons, those vaccinated in the current season only and not in the previous five seasons had the highest VE against influenza H3N2 and B, with progressively lower VE with increasing vaccines received over the previous five seasons [6]. Few studies reported on vaccination history beyond prior and current seasons, and they did not group history consistently, so further analysis incorporating the effects of serial vaccination from these studies was not possible, but is an important analysis to conduct in the future when more data are available. Second, our study did not account for past influenza infection, which may have provided some protective effect against laboratory-confirmed influenza in subsequent seasons [43]. A patient’s first exposure to influenza vaccination or infection can impact subsequent responses to vaccination or infection (referred to as original antigenic sin or back-boosting), which was not accounted for in this study [44]. Third, this study did not differentiate the types of influenza vaccines used for vaccination (e.g., live attenuated or inactivated; quadrivalent or trivalent; adjuvanted or unadjuvanted; high dose or standard dose). Given the differing types of immune response induced by these various products, different impacts of prior vaccination on current season VE may result. Fourth, we evaluated the absolute difference in VE, instead of looking at a ratio, which could be considered more appropriate given the scale on which VE is calculated. However, reporting ratios introduces other challenges, such as accommodating negative values and estimating confidence intervals. Since deriving practical conclusions for annual vaccine decision-making was the goal, we reported more intuitive differences in VE, as others have done previously [14, 45]. Finally, based on the limited available information in each study, we could not adjust for the match between the current season’s vaccine and the circulating strains, the prior season’s vaccine and the current season’s circulating strains, or changes in vaccine strains from one season to the other, all of which may affect VE from 1 year to the other as noted by Smith et al.’s antigenic distance hypothesis [46]. Skowronski et al. recently examined VE for influenza H3N2 in Canada using this framework and concluded the effects of repeated vaccination were consistent with the antigenic distance hypothesis [47]. We attempted to assess VE based on antigenic distance in the included articles by considering the vaccine strain and circulating strain match where possible, but not all studies provided detailed strain information. In the articles with sufficient information, the variation of vaccine and circulating strain matches were too few and were grouped by season, and as seasonal analysis was already included in our meta-analysis, no further information was gained. However, consistent with Skowronski et al.’s findings, we did find significant negative interference in the 2014–2015 influenza season, which supports the antigenic distance hypothesis which predicts that this would occur when vaccine strains are homologous from 1 year to the next but the prior season’s vaccine does not match the current circulating strain. Future VE studies should continue to incorporate vaccination status in prior seasons and provide as much detail as possible to allow assessment of the match between vaccine and circulating strains and the changes in vaccine strains over time. Future studies should also assess the impact of vaccination over multiple past seasons.

## Conclusions

In conclusion, from the patient’s perspective, vaccination in the current season is generally the best option regardless of prior season vaccination. From a policy perspective, although VE was lower against H3N2 and B for individuals vaccinated in both seasons compared to those vaccinated in the current season only, this result may vary from season to season; in addition, it should be noted that past vaccination history cannot be altered and this comparison disregards susceptibility to influenza during the prior season among those vaccinated in the current season only.

## Additional files

Additional file 1: Database Search Strategy (DOCX 20 kb)

Additional file 2: **Table S1.** Study characteristics of articles included in the meta-analysis and/or qualitative synthesis (DOCX 20 kb)

Additional file 3: **Figure S1.** Results of the risk of bias assessment by category using the Newcastle-Ottawa Scale (PDF 285 kb)

Additional file 4: **Table S2.** Meta-analysis pooled results by influenza season and influenza type/subtype (DOCX 26 kb)

## Abbreviations

CINAHL: Cumulative Index to Nursing and Allied Health Literature
NOS: Newcastle-Ottawa scale
PRISMA: Preferred Reporting Items for Systematic Reviews and Meta-Analysis
VE: Vaccine effectiveness
ΔVE: Absolute difference in vaccine effectiveness

## References

[1] World Health Organization (2012). Vaccines against influenza WHO position paper—November 2012. Wkly Epidemiol Rec.

[2] National Advisory Committee on Immunization (2017). (NACI): Canadian Immunization Guide Chapter on Influenza and Statement on Seasonal Influenza Vaccine for 2017–2018.

[3] Hoskins T, Davies J, Smith A, Miller C, Allchin A (1979). Assessment of inactivated influenza-A vaccine after three outbreaks of influenza A at Christ’s Hospital. Lancet.

[4] Keitel WA, Cate TR, Couch RB, Huggins LL, Hess KR (1997). Efficacy of repeated annual immunization with inactivated influenza virus vaccines over a five year period. Vaccine.

[5] Beyer WE, de Bruijn IA, Palache AM, Westendorp RG, Osterhaus AD. Protection against influenza after annually repeated vaccination: a meta-analysis of serologic and field studies. Arch Intern Med. 1999;159(2):182–8. 10.1001/archinte.159.2.182 9927102

[6] McLean HQ, Thompson MG, Sundaram ME, Meece JK, McClure DL, Friedrich TC, Belongia EA (2014). Impact of repeated vaccination on vaccine effectiveness against influenza A(H3N2) and B during 8 seasons. Clin Infect Dis.

[7] Skowronski DM, Chambers C, Sabaiduc S, De Serres G, Winter AL, Dickinson JA, Krajden M, Gubbay JB, Drews SJ, Martineau C, Eshaghi A, Kwindt TL, Bastien N, Li Y. A perfect storm: Impact of genomic variation and serial vaccination on low influenza vaccine effectiveness during the 2014-2015 season. Clin Infect Dis. 2016;63(1):21–32. 10.1093/cid/ciw176 PMC4901864 27025838

[8] Ohmit SE, Thompson MG, Petrie JG, Thaker SN, Jackson ML, Belongia EA, Zimmerman RK, Gaglani M, Lamerato L, Spencer SM, Jackson L, Meece JK, Nowalk MP, Song J, Zervos M, Cheng PY, Rinaldo CR, Clipper L, Shay DK, Piedra P, Monto AS (2014). Influenza vaccine effectiveness in the 2011-2012 season: protection against each circulating virus and the effect of prior vaccination on estimates. Clin Infect Dis.

[9] Moher D, Liberati A, Tetzlaff J, Altman DG, Prisma Group. Preferred reporting items for systematic reviews and meta-analyses: The PRISMA statement. PLoS Med. 2009;6(7):e1000097. 10.1371/journal.pmed.1000097 PMC2707599 19621072

[10] Wells G, Shea B, O’Connell D, Peterson J, Welch V, Losos M, Tugwell P. The Newcastle-Ottawa Scale (NOS) for assessing the quality of nonrandomised studies in meta-analyses. Ottawa: Ottawa Hospital Research Institute. http://www.ohri.ca/programs/clinical_epidemiology/oxford.asp.

[11] Lansbury LE, Smith S, Beyer W, Karamehic E, Pasic-Juhas E, Sikira H, Mateus A, Oshitani H, Zhao H, Beck CR. Effectiveness of 2009 pandemic influenza A (H1N1) vaccines: a systematic review and meta-analysis. Vaccine. 2017;35(16):1996–2006. 10.1016/j.vaccine.2017.02.059 28302409

[12] Domnich A, Arata L, Amicizia D, Puig-Barberà J, Gasparini R, Panatto D. Effectiveness of MF59-adjuvanted seasonal influenza vaccine in the elderly: a systematic review and meta-analysis. Vaccine.2017;35(4):513–20. 10.1016/j.vaccine.2016.12.011 28024956

[13] Carpenter J, Bithell J (2000). Bootstrap confidence intervals: when, which, what? A practical guide for medical statisticians. Stat Med.

[14] Feng S, Cowling BJ, Sullivan SG (2016). Influenza vaccine effectiveness by test-negative design–comparison of inpatient and outpatient settings. Vaccine.

[15] Castilla J, Moran J, Martinez-Artola V, Reina G, Martinez-Baz I, Cenoz MG, Alvarez N, Irisarri F, Arriazu M, Elia F. Effectiveness of trivalent seasonal and monovalent influenza A (H1N1) 2009 vaccines in population with major chronic conditions of Navarre, Spain: 2010/11 mid-season analysis. Euro Surveill. 2011;16(7). 21345321

[16] Castilla J, Navascués A, Fernández-Alonso M, Reina G, Pozo F, Casado I, Guevara M, Martínez-Baz I, Barricarte A, Ezpeleta C (2016). Effectiveness of subunit influenza vaccination in the 2014–2015 season and residual effect of split vaccination in previous seasons. Vaccine.

[17] Fu C, Xu J, Lin J, Wang M, Li K, Ge J, Thompson MG (2015). Concurrent and cross-season protection of inactivated influenza vaccine against A (H1N1) pdm09 illness among young children: 2012–2013 case–control evaluation of influenza vaccine effectiveness. Vaccine.

[18] Gaglani M, Pruszynski J, Murthy K, Clipper L, Robertson A, Reis M, Chung JR, Piedra PA, Avadhanula V, Nowalk MP, Zimmerman RK, Jackson ML, Jackson LA, Petrie JG, Ohmit SE, Monto AS, McLean HQ, Belongia EA, Fry AM, Flannery B (2016). Influenza vaccine effectiveness against 2009 pandemic influenza A(H1N1) virus differed by vaccine type during 2013-2014 in the United States. J Infect Dis.

[19] Jimenez-Jorge S, Savulescu C, Pozo F, De Mateo S, Casas I, Ledesma J, Larrauri A (2012). cycEVA study team: effectiveness of the 2010–11 seasonal trivalent influenza vaccine in Spain: cycEVA study. Vaccine.

[20] Martínez-Baz I, Martínez-Artola V, Reina G, Guevara M, Cenoz MG, Morán J, Irisarri F, Arriazu M, Albeniz E, Castilla J (2013). Effectiveness of the trivalent influenza vaccine in Navarre, Spain, 2010–2011: a population-based test-negative case–control study. BMC Public Health.

[21] McLean HQ, Thompson MG, Sundaram ME, Kieke BA, Gaglani M, Murthy K, Piedra PA, Zimmerman RK, Nowalk MP, Raviotta JM, Jackson ML, Jackson L, Ohmit SE, Petrie JG, Monto AS, Meece JK, Thaker SN, Clippard JR, Spencer SM, Fry AM, Belongia EA (2015). Influenza vaccine effectiveness in the United States during 2012-2013: variable protection by age and virus type. J Infect Dis.

[22] Ohmit SE, Petrie JG, Malosh RE, Johnson E, Truscon R, Aaron B, Martens C, Cheng C, Fry AM, Monto AS (2016). Substantial influenza vaccine effectiveness in households with children during the 2013–2014 influenza season, when 2009 pandemic influenza A (H1N1) virus predominated. J Infect Dis.

[23] Ohmit SE, Petrie JG, Malosh RE, Fry AM, Thompson MG, Monto AS (2015). Influenza vaccine effectiveness in households with children during the 2012-2013 season: assessments of prior vaccination and serologic susceptibility. J Infect Dis.

[24] Pebody R, Andrews N, Fleming D, McMenamin J, Cottrell S, Smyth B, Durnall H, Robertson C, Carman W, Ellis J (2013). Age-specific vaccine effectiveness of seasonal 2010/2011 and pandemic influenza A (H1N1) 2009 vaccines in preventing influenza in the United Kingdom. Epidemiol Infect.

[25] Rondy M, Launay O, Puig-Barbera J, Gefenaite G, Castilla J, de Gaetano Donati K, Galtier F, Hak E, Guevara M, Costanzo S, European hospital IVE network, Moren A. 2012/13 influenza vaccine effectiveness against hospitalised influenza A(H1N1)pdm09, A(H3N2) and B: estimates from a European network of hospitals. Euro Surveill. 2015;20(2):21011. 10.2807/1560-7917.es2015.20.2.21011 25613779

[26] Simpson CR, Lone N, Kavanagh K, Ritchie LD, Robertson C, Sheikh A, McMenamin J (2015). Trivalent inactivated seasonal influenza vaccine effectiveness for the prevention of laboratory confirmed influenza in a Scottish population 2000–2009. Euro Surveill.

[27] Skowronski DM, Janjua NZ, De Serres G, Sabaiduc S, Eshaghi A, Dickinson JA, Fonseca K, Winter A, Gubbay JB, Krajden M (2014). Low 2012–13 influenza vaccine effectiveness associated with mutation in the egg-adapted H3N2 vaccine strain not antigenic drift in circulating viruses. PLoS One.

[28] Skowronski DM, Janjua NZ, De Serres G, Winter AL, Dickinson JA, Gardy JL, Gubbay J, Fonseca K, Charest H, Crowcroft NS, Fradet MD, Bastien N, Li Y, Krajden M, Sabaiduc S, Petric M (2012). A sentinel platform to evaluate influenza vaccine effectiveness and new variant circulation, Canada 2010-2011 season. Clin Infect Dis.

[29] Skowronski DM, Janjua NZ, Sabaiduc S, De Serres G, Winter AL, Gubbay JB, Dickinson JA, Fonseca K, Charest H, Bastien N, Li Y, Kwindt TL, Mahmud SM, Van Caeseele P, Krajden M, Petric M (2014). Influenza A/subtype and B/lineage effectiveness estimates for the 2011-2012 trivalent vaccine: cross-season and cross-lineage protection with unchanged vaccine. J Infect Dis.

[30] Skowronski DM, Chambers C, Sabaiduc S, De Serres G, Winter AL, Dickinson JA, Gubbay J, Fonseca K, Charest H, Krajden M, Petric M, Mahmud SM, Van Caeseele P, Bastien N, Eshaghi A, Li Y (2015). Integrated sentinel surveillance linking genetic, antigenic, and epidemiologic monitoring of influenza vaccine-virus relatedness and effectiveness during the 2013-2014 influenza season. J Infect Dis.

[31] Smithgall M, Vargas CY, Reed C, Finelli L, LaRussa P, Larson EL, Saiman L, Stockwell MS. Influenza vaccine effectiveness in a low-income, urban community cohort. Clin Infect Dis. 2016;62(3):358–60. 10.1093/cid/civ867 PMC4706631 26420801

[32] Sullivan SG, Kelly H (2013). Stratified estimates of influenza vaccine effectiveness by prior vaccination: caution required. Clin Infect Dis.

[33] Syrjänen RK, Jokinen J, Ziegler T, Sundman J, Lahdenkari M, Julkunen I, Kilpi TM (2014). Effectiveness of pandemic and seasonal influenza vaccines in preventing laboratory-confirmed influenza in adults: a clinical cohort study during epidemic seasons 2009–2010 and 2010–2011 in Finland. PLoS One.

[34] Thompson MG, Clippard J, Petrie JG, Jackson ML, McLean HQ, Gaglani M, Reis EC, Flannery B, Monto AS, Jackson L (2016). Influenza vaccine effectiveness for fully and partially vaccinated children 6 months to 8 years old during 2011–2012 and 2012–2013. Pediatr Infect Dis J.

[35] Thompson MG, Li DK, Shifflett P, Sokolow LZ, Ferber JR, Kurosky S, Bozeman S, Reynolds SB, Odouli R, Henninger ML, Kauffman TL, Avalos LA, Ball S, Williams JL, Irving SA, Shay DK, Naleway AL (2014). Pregnancy and influenza project workgroup: effectiveness of seasonal trivalent influenza vaccine for preventing influenza virus illness among pregnant women: a population-based case-control study during the 2010-2011 and 2011-2012 influenza seasons. Clin Infect Dis.

[36] Valenciano M, Kissling E, Reuss A, Rizzo C, Gherasim A, Horvath JK, Domegan L, Pitigoi D, Machado A, Paradowska-Stankiewicz IA, Bella A, Larrauri A, Ferenczi A, Donell JO, Lazar M, Pechirra P, Korczynska MR, Pozo F, Moren A, I-MOVE multicentre case-control team. Vaccine effectiveness in preventing laboratory confirmed influenza in primary care patients in a season of co-circulation of influenza a(H1N1)pdm09, B and drifted A(H3N2), I-MOVE multicentre case-control study, Europe 2014/15. Euro Surveill. 2016;21(7). 10.2807/1560-7917.ES.2016.21.7.30139. 10.2807/1560-7917.ES.2016.21.7.30139 26924024

[37] Petrie JG, Ohmit SE, Cheng CK, Martin ET, Malosh RE, Lauring AS, Lamerato LE, Reyes KC, Flannery B, Ferdinands JM, Monto AS; Influenza Vaccine Effectiveness Against Antigenically Drifted Influenza Higher Than Expected in Hospitalized Adults: 2014–2015. Clin Infect Dis. 2016;63(8):1017–25. 10.1093/cid/ciw432. 10.1093/cid/ciw432 27369320

[38] Casado I, Domínguez A, Toledo D, Chamorro J, Force L, Soldevila N, Astray J, Egurrola M, Godoy P, Mayoral JM (2016). Effect of influenza vaccination on the prognosis of hospitalized influenza patients. Expert Rev Vaccines.

[39] Jackson ML, Nelson JC (2013). The test-negative design for estimating influenza vaccine effectiveness. Vaccine.

[40] Sullivan SG, Tchetgen Tchetgen EJ, Cowling BJ (2016). Theoretical basis of the test-negative study design for assessment of influenza vaccine effectiveness. Am J Epidemiol.

[41] Orenstein EW, De Serres G, Haber MJ, Shay DK, Bridges CB, Gargiullo P, Orenstein WA (2007). Methodologic issues regarding the use of three observational study designs to assess influenza vaccine effectiveness. Int J Epidemiol.

[42] Belongia EA, Skowronski DM, McLean HQ, Chambers C, Sundaram ME, De Serres G. Repeated annual influenza vaccination and vaccine effectiveness: review of evidence. Expert Rev Vaccines. 2017;16(7):1–14. 10.1080/14760584.2017.1334554 28562111

[43] Castilla J, Navascues A, Fernandez-Alonso M, Reina G, Albeniz E, Pozo F, Alvarez N, Martinez-Baz I, Guevara M, Garcia-Cenoz M, Irisarri F, Casado I, Ezpeleta C, Primary Health Care Sentinel Network and Network for Influenza Surveillance in Hospitals of Navarra. Effects of previous episodes of influenza and vaccination in preventing laboratory-confirmed influenza in Navarre, Spain, 2013/14 season. Euro Surveill. 2016;20(22). 10.2807/1560-7917.ES.2016.21.22.30243.

[44] Francis T (1960). On the doctrine of original antigenic sin. Proc Am Philos Soc.

[45] Leung VK, Cowling BJ, Feng S, Sullivan SG. Concordance of interim and final estimates of influenzavaccine effectiveness: a systematic review. Euro Surveill. 2016;21(16). 10.2807/1560-7917.ES.2016.21.16.30202. 10.2807/1560-7917.ES.2016.21.16.30202 27124573

[46] Smith DJ, Forrest S, Ackley DH, Perelson AS (1999). Variable efficacy of repeated annual influenza vaccination. Proc Natl Acad Sci U S A.

[47] Skowronski DM, Chambers C, De Serres G, Sabaiduc S, Winter AL, Dickinson JA, Gubbay JB, Fonseca K, Drews SJ, Charest H, Martineau C, Krajden M, Petric M, Bastien N, Li Y, Smith DJ. Serial vaccination and the antigenic distance hypothesis: effects on influenza vaccine effectiveness during A(H3N2) epidemics in Canada, 2010-11 to 2014-15. J Infect Dis. 2017;215(7):1059-99. 10.1093/infdis/jix074 PMC5853783 28180277

