# Supplementary material for: The impact of repeated vaccination on influenza vaccine effectiveness: a systematic review and meta-analysis
Source: BMC Med. 2019 Jan 10;17:9. doi: 10.1186/s12916-018-1239-8 (PMC6327561; doi:10.1186/s12916-018-1239-8)
Supplement: Supplementary file 1 — Database Search Strategy (DOCX 20 kb) [file 12916_2018_1239_MOESM1_ESM.docx]

**Additional file 1- Database Search Strategy**

## Databases

1. MEDLINE
2. Embase
3. Cumulative Index to Nursing and Allied Health Literature (CINAHL)
4. PubMed

## Search strategies

**Ovid MEDLINE(R) In-Process & Other Non-Indexed Citations and Ovid MEDLINE(R)**

| # | Searches | Results |
| --- | --- | --- |
| 1 | Influenza, Human/ or exp Influenza A Virus/ or exp Influenza B virus/ or Influenza Vaccines/ or ((flu or influenza).ti,kw,kf. and ("in data review" or in process or "pubmed not medline").st.) | 67821 |
| 2 | Vaccines/ or Viral Vaccines/ or Vaccines, Attenuated/ or Vaccines, Inactivated/ or Vaccination/ or Mass Vaccination/ or Immunization/ or Immunization, Secondary/ or Immunization Programs/ or Influenza Vaccines/ or ((vaccin* or revaccinat* or immunis* or immuniz*).ti,kw,kf. and ("in data review" or in process or "pubmed not medline").st.) | 176751 |
| 3 | (effectiveness or impact or efficacy or efficacious or protection or protective or performance).ti,kw,kf. or (effectiveness or impact or efficacy or efficacious or protection or protective or performance).ab. /freq=2 or ((vaccin* or revaccinat* or immunis* or immuniz*) adj3 (effectiveness or impact or efficacy or efficacious or protection or protective or performance)).ab. or vaccine effectiveness.mp. | 1013209 |
| 4 | Influenza, Human/ep or Influenza, Human/sn or Disease Outbreaks/ or Epidemiological Monitoring/ or Hospitalization/ or Incidence/ or Morbidity/ or Epidemiology/ or Numbers Needed To Treat/ or Population Surveillance/ or Public Health Surveillance/ or Sentinel Surveillance/ or Prevalence/ or Seasons/ or Models, Immunological/ or exp North America/ep or exp Australia/ep or exp Cities/ep or exp Europe/ep or "vaccine effectiveness".mp. or (outbreak* or hospitaliz* or hospitalis* or surveillance or morbidity or incidence or prevalence or "herd immunity" or "herd effect" or "herd protection" or epidemiolog* or model* or season* or ((flu or influenza or disease* or infect* or illness*) adj3 (cases or rate*)) or ((prevent* or reduc* or decreas* or avert* or fewer or less) adj10 (flu or influenza or disease* or infect* or illness* or cases or rate*)) or "attack rate*").ti,kw,kf. or (outbreak* or hospitaliz* or hospitalis* or surveillance or morbidity or incidence or prevalence or "herd immunity" or "herd effect" or "herd protection" or epidemiolog* or model* or season* or ((flu or influenza or disease* or infect* or illness*) adj3 (cases or rate*)) or ((prevent* or reduc* or decreas* or avert* or fewer or less) adj10 (flu or influenza or disease* or infect* or illness* or cases or rate*)) or "attack rate*").ab. /freq=2 | 2547206 |
| 5 | 1 and 2 and 3 and 4 | 3107 |
| 6 | (exp Animals/ not Humans/) or Animals/ or ((mouse or mice or macaque* or ferret* or animal* or bird* or poultry or chicken* or swine or pig* or duck*).ti. and ("in data review" or in process or "pubmed not medline").st.) | 5908214 |
| 7 | 5 not 6 | 2413 |
| 8 | limit 7 to english | 2146 |
| 9 | remove duplicates from 8 | 2096 |

**Embase**

| # | Searches | Results |
| --- | --- | --- |
| 1 | *influenza/ or *seasonal influenza/ or exp *influenza A/ or exp *influenza B/ or *influenza virus/ or exp *influenza virus a/ or exp *influenza virus b/ or *influenza vaccine/ or *influenza vaccination/ | 59756 |
| 2 | *immunization/ or *inactivated vaccine/ or *influenza vaccination/ or *influenza vaccine/ or *live vaccine/ or *mass immunization/ or *revaccination/ or *vaccination/ or *vaccine/ or *virus vaccine/ | 119270 |
| 3 | (effectiveness or impact or efficacy or efficacious or protection or protective or performance).ti,kw. or (effectiveness or impact or efficacy or efficacious or protection or protective or performance).ab. /freq=2 or ((vaccin* or revaccinat* or immunis* or immuniz*) adj3 (effectiveness or impact or efficacy or efficacious or protection or protective or performance)).ab. or vaccine effectiveness.mp. | 1380175 |
| 4 | *influenza/ep or *seasonal influenza/ep or disease surveillance/ or sentinel surveillance/ or numbers needed to treat/ or epidemic/ or epidemiological data/ or epidemiological monitoring/ or epidemiology/ or hospitalization/ or incidence/ or infection rate/ or morbidity/ or prevalence/ or seasonal variation/ or North America/ep or exp Australia/ep or exp Europe/ep or "vaccine effectiveness".mp. or (outbreak* or hospitaliz* or hospitalis* or surveillance or morbidity or incidence or prevalence or "herd immunity" or "herd effect" or "herd protection" or epidemiolog* or model* or season* or ((flu or influenza or disease* or infect* or illness*) adj3 (cases or rate*)) or ((prevent* or reduc* or decreas* or avert* or fewer or less) adj10 (flu or influenza or disease* or infect* or illness* or cases or rate*)) or "attack rate*").ti,kw. or (outbreak* or hospitaliz* or hospitalis* or surveillance or morbidity or incidence or prevalence or "herd immunity" or "herd effect" or "herd protection" or epidemiolog* or model* or season* or ((flu or influenza or disease* or infect* or illness*) adj3 (cases or rate*)) or ((prevent* or reduc* or decreas* or avert* or fewer or less) adj10 (flu or influenza or disease* or infect* or illness* or cases or rate*)) or "attack rate*").ab. /freq=2 | 3470043 |
| 5 | 1 and 2 and 3 and 4 | 3069 |
| 6 | (exp animal/ not human/) or animal/ | 5050017 |
| 7 | 5 not 6 | 2664 |
| 8 | limit 7 to english language | 2384 |
| 9 | limit 8 to embase | 2173 |
|  | | |

**CINAHL Plus with Full Text**

| # | Query | Results |
| --- | --- | --- |
| S1 | (MH "Influenza A Virus+") OR (MH "Influenza B Virus") OR (MH "Influenza+") OR (MH "Influenza, Human") OR (MH "Influenza, Seasonal") OR (MH "Influenza Vaccine") | 18,952 |
| S2 | (MH "Vaccines") OR (MH "Viral Vaccines") OR (MH "Immunization") OR (MH "Immunization Programs") OR (MH "Immunization, Secondary") OR (MH "Influenza Vaccine") | 29,998 |
| S3 | TI(effectiveness OR impact OR efficacy OR efficacious OR protection OR protective OR performance) OR AB(effectiveness OR impact OR efficacy OR efficacious OR protection OR protective OR performance) OR "vaccine effectiveness" | 406,626 |
| S4 | (MM "Influenza, Human/EP") OR (MM "Influenza/EP") OR (MH "Disease Outbreaks") OR (MH "Epidemiological Research") OR (MH "Epidemiology") OR (MH "Population Surveillance") OR (MH "Disease Surveillance") OR (MH "Hospitalization") OR (MH "Morbidity") OR (MH "Incidence") OR (MH "Prevalence") OR (MH "Seasons") OR (MW "epidemiology") OR "vaccine effectiveness" OR TI(outbreak* OR hospitaliz* OR hospitalis* OR surveillance OR morbidity OR incidence OR prevalence OR epidemiolog* OR "herd immunity" OR "herd effect" OR "herd protection" OR ((flu or influenza or disease* or infect* or illness*) N3 (cases or rate*)) OR ((prevent* or reduc* or decreas* or avert* or fewer or less) N10 (flu or influenza or disease* or infect* or illness* or cases or rate*)) OR "attack rate*") OR AB(outbreak* OR hospitaliz* OR hospitalis* OR surveillance OR morbidity OR incidence OR prevalence OR epidemiolog* OR "herd immunity" OR "herd effect" OR "herd protection" OR ((flu or influenza or disease* or infect* or illness*) N3 (cases or rate*)) OR ((prevent* or reduc* or decreas* or avert* or fewer or less) N10 (flu or influenza or disease* or infect* or illness* or cases or rate*)) OR "attack rate*") | 573,334 |
| S5 | S1 AND S2 AND S3 AND S4 | 745 |
| S6 | S5 AND LA English | 739 |
| S7 | ( (MH "Animals+") NOT (MH "Human") ) OR (MH "Animals" ) | 65,946 |
| S8 | S6 NOT S7 | 728 |

**PubMed**

| # | Query | Results |
| --- | --- | --- |
| #1 | ("Influenza, Human"[MeSH:NoExp] OR "Influenza A Virus"[MeSH] OR "Influenza B virus"[MeSH] OR "Influenza Vaccines"[MeSH:NoExp] OR flu[TI] or influenza[TI] OR flu[OT] or influenza[OT]) AND ("Vaccines"[MeSH:NoExp] OR "Viral Vaccines"[MeSH:NoExp] OR "Vaccines, Attenuated"[MeSH:NoExp] OR "Vaccines, Inactivated"[MeSH:NoExp] OR "Vaccination"[MeSH:NoExp] OR "Mass Vaccination"[MeSH:NoExp] OR "Immunization"[MeSH:NoExp] OR "Immunization, Secondary"[MeSH:NoExp] OR "Immunization Programs"[MeSH:NoExp] OR "Influenza Vaccines"[MeSH:NoExp] OR vaccin*[TI] OR revaccinat*[TI] OR immunis*[TI] OR immuniz*[TI] OR vaccin*[OT] OR revaccinat*[OT] OR immunis*[OT] OR immuniz*[OT]) AND (effectiveness[TI] OR impact[TI] OR efficacy[TI] OR efficacious[TI] OR protection[TI] OR protective[TI] OR performance[TI] OR effectiveness[OT] OR impact[OT] OR efficacy[OT] OR efficacious[OT] OR protection[OT] OR protective[OT] OR performance[OT] OR "vaccine effectiveness"[TW]) AND (publisher[SB] OR inprocess[SB] OR pubmednotmedline[SB] OR pubstatusaheadofprint) AND English[LA] | 260 |
