# Supplementary material for: The impact of repeated vaccination on influenza vaccine effectiveness: a systematic review and meta-analysis
Source: BMC Med. 2019 Jan 10;17:9. doi: 10.1186/s12916-018-1239-8 (PMC6327561; doi:10.1186/s12916-018-1239-8)
Supplement: Supplementary file 2 — Table S1. Study characteristics of articles included in the meta-analysis and/or qualitative synthesis (DOCX 20 kb) [file 12916_2018_1239_MOESM2_ESM.docx]

**Table S1.** Study characteristics of articles included in the meta-analysis and/or qualitative synthesis

| **Author, Publication Year** | **Participant Recruitment** | **Case definition** | **Adjustment Variables** |
| --- | --- | --- | --- |
| Jimenez-Jorge et al., 2012 | Spanish Influenza Sentinel Surveillance System patients with ARI, first 2 patients <65 swabbed, all patients ≥65 | Sudden onset of symptoms and at least one of: fever or feverishness, malaise, headache, myalgia, and at least one of: cough, sore throat, or shortness of breath | Age group, and week of swabbing |
| Martinez-Baz et al., 2013 | Primary care physician patients with ILI | Sudden onset of any general symptom (fever, feverishness, malaise, headache or myalgia) and any respiratory symptom (cough, sore throat, shortness of breath) | Sex, age, major chronic conditions, outpatient visits in the previous year, swabbing within 4 days of symptom onset, health care setting and period |
| Skowronski et al., 2012 | Sentinel outpatient clinics patients with ILI | Acute onset of fever and cough and ≥1 of sore throat, arthralgia, myalgia or prostration | Not clearly reported |
| Syrjanen et al., 2014 | Voluntary enrolment | Sudden onset of the following self reported clinical signs and symptoms: measured fever (≥38°C) and at least one sign or symptom of acute respiratory infection. In addition, pneumonia diagnosed by a physician was also regarded as an ILI. | Age group, gender, underlying medical condition, pregnancy |
| Fu et al., 2015 | Hospital-based outpatients with ILI | History of fever with cough or sore throat | Conditional logistic - matched to those without ILI on gender, age (months) and neighbourhood |
| Gaglani et al., 2016 | US Flu Network patients with ARI | Cough and onset of illness <7 days before enrolment | Site, age, calendar time, any high-risk condition, sex, race/ethnicity, general health status, and interval from illness onset to enrolment |
| Ohmit et al., 2016 | Recruited households with at least 4 members (minimum 2 children) | ARI in which two of the following symptoms were present: cough, fever or feverishness, nasal congestion, chills, headache, body aches, and/or sore throat | Age in months (natural cubic spline) and medical record-documented high-risk health status |
| Thompson et al., 2014 | Incentive offered to members of Kaiser Permanente with at least one prenatal visit | Medically-attended ARI (International Classification of Disease- codes 460-466, 480-488) | Age, race, ethnicity (Hispanic), and high risk medical condition, site, season, trimester, illness medically attended, days between illness onset and swab |
| Skowronski et al., 2014b | Sentinel outpatient clinics patients with ILI | Acute onset of fever and cough and ≥1 of sore throat, arthralgia, myalgia or prostration; for 2011-12, fever was not required in the elderly ≥65 years old | Age, comorbidity, province, interval, and week |
| Rondy et al., 2015 | Hospital patients admitted with ILI | ILI symptoms (one systemic, one respiratory symptom) within the past seven days | Study site, month of symptom onset, age, and comorbidities |
| Skowronski et al., 2014a | Sentinel outpatient clinics patients with ILI | Acute onset of fever and cough and ≥1 of sore throat, arthralgia, myalgia or prostration; Fever is not required for elderly patients ≥65 years old | Age, comorbidity, province, interval, week |
| Valenciano et al., 2016 | Patients presenting with ILI | Included patients who presented more than 14 days after the start of the national vaccination campaign and who met European Union influenza-like illness case definition | Age (restricted cubic spline or age group), onset date (restricted cubic spline), sex, chronic condition, and study site |
| Pebody et al., 2013 | Primary care patients with ILI | ARI with fever or complaint of feverishness | Age group, gender, time period, and surveillance scheme |
| Skowronski et al., 2015 | Outpatient patients with ILI | Acute onset of fever and cough and ≥1 of sore throat, arthralgia, myalgia or prostration; Fever is not required for elderly patients ≥65 years old | Age group, comorbidity, province, specimen collection interval, and calendar-time |
| McLean et al., 2014 | Eligible individuals actively recruited during a clinical encounter for ARI | ARI | Age, sex, high-risk conditions, season, interval (days) from onset to sample collection, and influenza diagnosis in previous seasons |
| McLean et al., 2015 | Outpatient patients with ARI | ARI with cough | Network site, subject age, presence of high-risk conditions, calendar time |
| Thompson et al., 2016 | Outpatient patients with ARI | ARI (onset ≥7 days) with cough | Study site, month of enrolment, age (in months), high-risk status, race/ethnicity and days from illness onset to enrolment. H3N2 model also included variable for season and an interaction term for season by month |
| Skowronski et al., 2016 | Sentinel outpatient clinics patients with ILI | Acute onset of fever and cough and ≥1 of sore throat, arthralgia, myalgia or prostration; Fever is not required for elderly patients ≥65 years old | Age group, sex, comorbidity, province, specimen collection interval and calendar-time |
| Simpson et al., 2015 | Patient-level data linked to virology dataset to identify confirmed influenza | Not available | Not clearly reported |
| Castilla et al., 2011 | Hospitalized and outpatient patients with ILI or ARI, limited to patients with major chronic condition | Medically attended ILI | Sex, age (1-14 years; 15-59 years; 65 years and older), children in the household, urban/rural residence, healthcare setting (primary health care, emergency room, hospitalization) and date (week 43-49 2010; week 50 2010 - week 1 2011; week 2-3 2011) |
| Ohmit et al., 2014 | Ambulatory and urgent care patients with ARI | ARI with cough and or fever/feverishness of <7 days duration | Network centre, subject age in months, sex, race/ethnicity categories, presence of high-risk health conditions, self-rated health status, time (days) between illness onset and specimen collection, and calendar time |
| Ohmit et al., 2015 | Recruited households with at least 4 members (minimum 2 children) | ARI in which two of the following symptoms were present: cough, fever or feverishness, nasal congestion, chills, headache, body aches, and/or sore throat | Age in months (natural cubic spline) and documentation (present or absent) of high-risk health status in the medical record |
| Smithgall et al., 2016 | 275 selected households enrolled in larger study on ARI | ARI symptoms (rhinorrhea/congestion, pharyngitis, cough, body aches or feverishness) | Age group, sex, chronic respiratory conditions |
| Castilla et al., 2016 | Hospitalized and outpatient patients with ILI | Sudden onset of any general symptom (fever or feverishness, malaise, headache or myalgia) in addition to any respiratory symptom (cough, sore throat or shortness of breath). Nasopharyngeal and pharyngeal swabs from all patients with ILI whose symptoms began less than 5 days previously | Sex, age group (<5, 5–14, 15–44, 45–64, 65–84, and ≥85 years), any major chronic condition, three-week period of sample collection, and healthcare setting (primary healthcare and hospital) |
| Sullivan & Kelly, 2013 | Not reported | Not reported | Age group, high-risk health status, week, and time between onset and polymerase chain reaction request |
| Petrie et al., 2016 | Hospitalized patients with ARI identified using Electronic Medical Records | ILI (e.g. influenza, respiratory infection, cough, bronchitis), pneumonia, and exacerbations of asthma or chronic obstructive pulmonary disease; patients with other diagnoses, including respiratory distress, shortness of breath, and acute exacerbations of other chronic respiratory conditions (e.g. congestive heart failure) were also eligible if evidence of an ARI (e.g. new or worsening cough) was included in the admission note. | Age, sex, hospital, calendar time, time from illness, onset to specimen collection, frailty score, and Charlson Comorbidity Index |

**Abbreviations:** ARI = acute respiratory illness; ILI=Influenza-like illness
