# Supplementary figures and images for: The impact of repeated vaccination on influenza vaccine effectiveness: a systematic review and meta-analysis
Source: BMC Med. 2019 Jan 10;17:9. doi: 10.1186/s12916-018-1239-8 (PMC6327561; doi:10.1186/s12916-018-1239-8)

**Figure S1.** Results of the risk of bias assessment by category using the Newcastle-Ottawa Scale

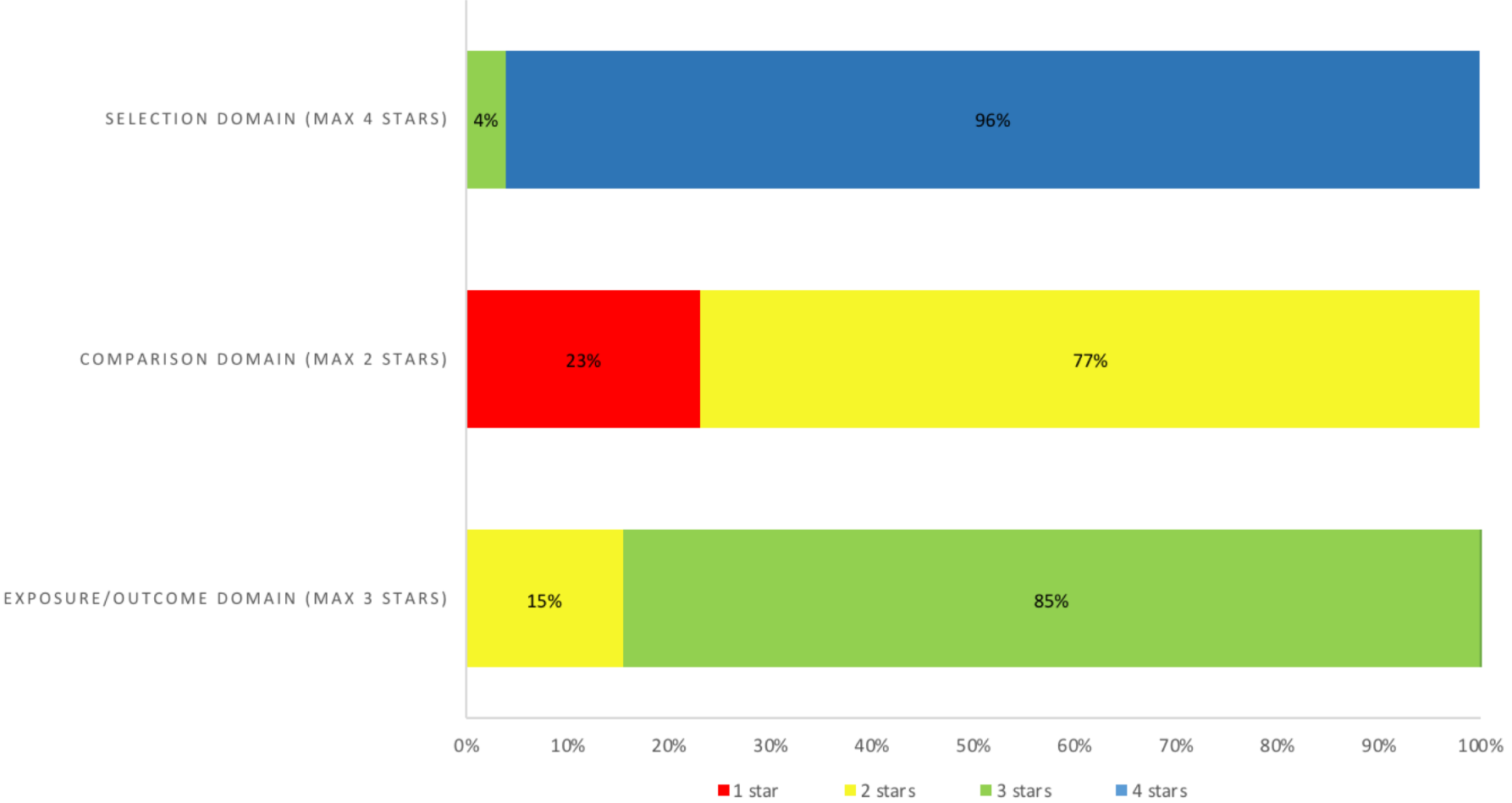

Supplement: Supplementary file 3 — Figure S1. Results of the risk of bias assessment by category using the Newcastle-Ottawa Scale (PDF 285 kb) [file 12916_2018_1239_MOESM3_ESM.pdf]
