# Supplementary material for: The impact of repeated vaccination on influenza vaccine effectiveness: a systematic review and meta-analysis
Source: BMC Med. 2019 Jan 10;17:9. doi: 10.1186/s12916-018-1239-8 (PMC6327561; doi:10.1186/s12916-018-1239-8)
Supplement: Supplementary file 4 — Table S2. Meta-analysis pooled results by influenza season and influenza type/subtype (DOCX 26 kb) [file 12916_2018_1239_MOESM4_ESM.docx]

**Table S2.** Meta-analysis pooled results by influenza season and influenza type/subtype

| Vaccinated Both Seasons Versus Vaccinated Prior Season Only | | | | | |
| --- | --- | --- | --- | --- | --- |
| **H1N1** | Influenza Season | Number of estimates (Number of studies) | ΔVE* (95% CI)  VE_both_ – VE_prior only_ | I^2†^ | Cochran’s Q (degrees of freedom, p value) |
|  | 2010-11 | 5 (5) | 19% (–4%, 42%) | 0 | χ^2^=1.20 (d.f.=4, p=0.88) |
|  | 2011-12 | 1 (1) | 30% (–6%, 88%) | n/a | n/a |
|  | 2012-13 | 4 (2) | 32% (16%, 47%) | 0 | χ^2^=0.58 (d.f.=3, p=0.90) |
|  | 2013-14 | 4 (3) | 16% (–5%, 36%) | 0 | χ^2^=0.80 (d.f.=3, p=0.85) |
|  | 2014-15 | 1 (1) | 55% (0%, 140%) | n/a | n/a |
|  | Multiple^§^ | 1 (1) | -21% (–66%, 84%) | n/a | n/a |
|  | **OVERALL** | **16 (13)** | **25% (14%, 35%)** | **0** | **χ^2^=6.39 (d.f.=15, p=0.97)** |
| **H3N2** | 2007-08 | 1 (1) | 10% (–28%, 56%) | n/a | n/a |
|  | 2011-12 | 2 (2) | 21% (–17%, 60%) | 0 | χ^2^=0.40 (d.f.=1, p=0.53) |
|  | 2012-13 | 6 (3) | 11% (–20%, 43%) | 44 | χ^2^=8.87 (d.f.=5, p=0.11) |
|  | 2014-2015 | 3 (3) | -17% (–53%, 18%) | 0 | χ^2^=1.70 (d.f.=2, p=0.43) |
|  | Multiple^§^ | 2 (2) | 14% (–15%, 42%) | 0 | χ^2^=0.001 (d.f.=1, p=0.94) |
|  | **OVERALL** | **14 (11)** | **7% (–7%, 21%)** | **4** | **χ^2^=13.5 (d.f.=13, p=0.41)** |
| **B** | 2010-11 | 1 (1) | 57% (15%, 107%) | n/a | n/a |
|  | 2011-12 | 1 (1) | 10% (–23%, 49%) | n/a | n/a |
|  | 2012-13 | 7 (4) | 23% (3%, 42%) | 0 | χ^2^=3.72 (d.f.=6, p=0.72) |
|  | 2013-14 | 1 (1) | 36% (–6%, 107%) | n/a | n/a |
|  | 2014-15 | 2 (2) | 25% (–14%, 65%) | 0 | χ^2^=0.74 (d.f.=1, p=0.39) |
|  | Multiple^§^ | 1(1) | -12% (–30%, 9%) | n/a | n/a |
|  | **OVERALL** | **13**^‡^ **(10)** | **18% (3%, 33%)** | **26** | **χ^2^=16.27 (d.f.=12, p=0.18)** |
| Vaccinated Current Season Only Versus Vaccinated Neither Season (reference group) | | | | | |
| **H1N1** | Influenza Season | Number of estimates (Number of studies) | Pooled VE_current only_ | I^2†^ | Cochran’s Q (degrees of freedom, p value) |
|  | 2010-11 | 5 (5) | 55% (25%, 73%) | 39 | χ^2^=6.58 (d.f.=4, p=0.16) |
|  | 2011-12 | 1 (1) | 73% (–16%, 94%) | n/a | n/a |
|  | 2012-13 | 4 (2) | 66% (32%, 83%) | 68 | χ^2^=9.32 (d.f.=3, p=0.03) |
|  | 2013-14 | 4 (3) | 65% (50%, 75%) | 0 | χ^2^=1.77 (d.f.=3, p=0.62) |
|  | 2014-15 | 1 (1) | 47% (–7%, 74%) | n/a | n/a |
|  | Multiple^§^ | 1 (1) | 68% (10%, 88%) | n/a | n/a |
|  | **Overall** | **16 (13)** | **62% (51%, 70%)** | **26** | **χ^2^=20.35 (d.f.=15, p=0.16)** |
| **H3N2** | 2007-08 | 1 (1) | 53% (23%, 72%) | n/a | n/a |
|  | 2011-12 | 2 (2) | 50% (23%, 68%) | 0 | χ^2^=0.74 (d.f.=1, p=0.39) |
|  | 2012-13 | 6 (3) | 38% (19%, 53%) | 12 | χ^2^=5.68 (d.f.=5, p=0.34) |
|  | 2014-2015 | 3 (3) | 48% (28%, 62%) | 0 | χ^2^=0.35 (d.f.=2, p=0.84) |
|  | Multiple^§^ | 2 (2) | 50% (11%, 71%) | 20 | χ^2^=1.25 (d.f.=1, p=0.26) |
|  | **OVERALL** | **14 (11)** | **45% (35%, 53%)** | **0** | **χ^2^=10.14 (d.f.=13, p=0.68)** |
| **B** | 2010-11 | 1 (1) | 56% (29%, 73%) | n/a | n/a |
|  | 2011-12 | 1 (1) | 52% (–8%, 78%) | n/a | n/a |
|  | 2012-13 | 7 (4) | 67% (55%, 77%) | 0 | χ^2^=2.73 (d.f.=6, =0.84) |
|  | 2013-14 | 1 (1) | 90% (58%, 98%) | n/a | n/a |
|  | 2014-15 | 2 (2) | 61% (35%, 76%) | 0 | χ^2^=0.03 (d.f.=2, p=0.86) |
|  | Multiple^§^ | 2 (2) | 67% (52%, 77%) | 0 | χ^2^=0.18 (d.f.=1, p=0.67) |
|  | **OVERALL** | **14 (11)** | **64% (57%, 71%)** | **0** | **χ^2^=7.47 (d.f.=13, p=0.88)** |
| Vaccinated Both Seasons Versus Vaccinated Current Season Only | | | | | |
| **H1N1** | Influenza Season | Number of estimates (Number of studies) | ΔVE* (95% CI)  VE_both_ – VE_current only_ | I^2†^ | Cochran’s Q (degrees of freedom, p value) |
|  | 2010-11 | 5 (5) | 12% (–11%, 34%) | 0 | χ^2^=0.73 (d.f.=4, p=0.95) |
|  | 2011-12 | 1 (1) | 13% (–20%, 103%) | n/a | n/a |
|  | 2012-13 | 4 (2) | 3% (–13%, 20%) | 0 | χ^2^=0.44 (d.f.=3, p=0.93) |
|  | 2013-14 | 4 (3) | -8% (–30%, 14%) | 0 | χ^2^=0.16 (d.f.=3, p=0.98) |
|  | 2014-15 | 1 (1) | 6% (–34%, 62%) | n/a | n/a |
|  | Multiple^§^ | 1 (1) | -1% (–50%, 57%) | n/a | n/a |
|  | **OVERALL** | **16 (13)** | **3% (–8%, 13%)** | **0** | **χ^2^=3.03 (**d.f.=**15, p=1.0)** |
| **H3N2** | 2007-08 | 1 (1) | -14% (–47%, 22%) | n/a | n/a |
|  | 2011-12 | 2 (2) | -19% (–63%, 25%) | 13 | χ^2^=1.15 (d.f.=1, p=0.28) |
|  | 2012-13 | 6 (3) | -8% (–33%, 16%) | 24 | χ^2^=6.61 (d.f.=5, p=0.25) |
|  | 2014-2015 | 3 (3) | -54% (–88%, –20%) | 29 | χ^2^=2.82 (d.f.=2, p=0.24) |
|  | Multiple^§^ | 2 (2) | **–**4% (–32%, 24%) | 0 | χ^2^=0.10 (d.f.=1, p=0.75) |
|  | **OVERALL** | **14 (11)** | **–20% (–36%, –4%)** | **35** | **χ^2^=20.07 (**d.f.=**13, p=0.75)** |
| **B** | 2010-11 | 1 (1) | –3% (–34%, 29%) | n/a | n/a |
|  | 2011-12 | 1 (1) | 7% (–30%, 67%) | n/a | n/a |
|  | 2012-13 | 7 (4) | –6% (–21%, 8%) | 0 | χ^2^=3.37 (d.f.=6, p=0.76) |
|  | 2013-14 | 1 (1) | –25% (–49%, 13%) | n/a | n/a |
|  | 2014-15 | 2 (2) | –20% (–48%, 8%) | 0 | χ^2^=0.15 (d.f.=1, p=0.70) |
|  | Multiple^§^ | 2 (2) | –15% (–32%, 3%) | 0 | χ^2^=0.08 (d.f.=2, p=0.78) |
|  | **OVERALL** | **14 (11)** | **–11% (–20%, –2 %)** | **0** | **χ^2^=6.11 (d.f.=13, p=0.94)** |

*ΔVE > 0 implies higher vaccine effectiveness estimate when vaccinated in both seasons

^†^No measure of heterogeneity was provided when only one estimate was available in a season

^‡^One study had no cases for prior season vaccination only, and so only 13 vaccine effectiveness estimates were included in this comparison

^§^Comparisons occurred across multiple seasons
